# Catamenial pneumothorax presenting as tension pneumothorax: a case report

**DOI:** 10.3389/frph.2026.1871134

**Published:** 2026-09-29

**Authors:** John Olukorode, Nwachukwu Nwachukwu, Akinboade Popoola, Heritage Kristilere, Yetunde Oladipupo, Rolake Akin-Adewale, Ayotemi Oluwagbemi, Oluwafemi Fafiola, Lucky Ogidan, Raymond Omiko

**Affiliations:** 1 Babcock University College of Health and Medical Sciences, Ilishan-Remo, Nigeria; 2 Department of Medicine, Darlington Memorial Hospital, Darlington, United Kingdom; 3 University of Lagos College of Medicine, Lagos, Nigeria; 4 University College Hospital Ibadan, Ibadan, Nigeria; 5 University of Ilorin Teaching Hospital, Ilorin, Nigeria; 6 Bowen University Teaching Hospital, Iwo, Nigeria; 7 Obafemi Awolowo University College of Health Sciences, Ife, Nigeria

**Keywords:** catamenial pneumothorax, endometriosis, pneumothorax, tension pneumothorax, thoracic endometriosis

## Abstract

**Introduction:**

Catamenial pneumothorax is a rare condition. It may present with complications such as tension pneumothorax.

**Case presentation:**

A 35-year-old nulliparous woman without a pre-existing diagnosis of endometriosis presented with recurrent episodes of chest tightness, breathlessness, and dizziness, occurring in relation to her menstrual cycle. Initial evaluation revealed a right-sided pneumothorax, requiring chest tube insertion. Although she experienced temporary improvement, symptoms recurred at the onset of her next menstrual period, with imaging confirming tension pneumothorax. Video-assisted thoracoscopic surgery (VATS) identified diaphragmatic perforations and endometriotic lesions.

**Conclusion:**

Timely diagnosis and treatment should be adopted in order to limit complications associated with catamenial pneumothorax.

## Introduction

Historically, catamenial pneumothorax (CP) was considered a rare syndrome, primarily attributed to intra-thoracic endometriosis. Despite being identified many years ago, its pathophysiology remains poorly understood. CP is defined as spontaneous pneumothorax occurring within 72 h before or after the onset of menstruation, although symptoms may occur 7 days before or up to 7 days after menstruation (1, 2). It is the most prevalent and widely recognised manifestation of Thoracic endometriosis syndromes, others being Catamenial haemothorax, Catamenial hemopneumothorax, Catamenial haemoptysis and endometriotic lung nodule. The aetiology of CP remains unknown, despite various proposed theories (3).

CP is likely underdiagnosed in many low resource settings in Africa. The incidence of catamenial and/or endometriosis-related pneumothorax is notably higher among nulliparous women of reproductive age. It accounts for one-third of all spontaneous pneumothorax cases in women (4).

Most cases of catamenial pneumothorax are mild, with symptoms typically resolving after menstruation. However, rare cases may present with tension pneumothorax, a potentially life-threatening complication. Such presentations remain underreported, particularly in African settings. This case report describes a woman in her 30 s who developed tension pneumothorax as a complication of catamenial pneumothorax, highlighting the need for heightened clinical vigilance and comprehensive management strategies.

## Case presentation

### Patient information

A 35-year-old nulliparous woman presented to the outpatient department with a 3-day history of chest tightness, exertional dyspnoea, and dizziness. Notably, symptom onset occurred 2 days prior to the onset of her menstrual period. She is not a known asthmatic and denied any prior similar episodes. Her past medical history was unremarkable, with no known chronic illnesses. No history of chronic lung disease in her family. Surgical history was significant for a myomectomy performed 4 years prior. Her menstrual cycles were regular, and she reported no history of dysmenorrhea, dyspareunia or chronic pelvic pain.

### Clinical findings

On examination, she was dyspneic but hemodynamically stable, with an oxygen saturation of 97% in room air. Chest examination revealed reduced breath sounds over the right hemithorax. Abdominal examination showed no mass and no tenderness. Other systemic examinations were unremarkable.

### Diagnostic assessment

Baseline investigations included a packed cell volume of 43%, normal WBC count of 6,000 (58% neutrophils, 30.2% lymphocytes) and platelet count of 230,000. Electrolytes, urea and creatinine levels were within normal limits. Liver function test and clotting profile were also normal. Erythrocyte sedimentation rate (ESR) was 4 mm/h, and viral markers were all negative. A chest radiograph demonstrated a right-sided pneumothorax with associated compressive atelectasis of the right lung (Figure 1). Based on the temporal association with her menstrual cycle, a provisional diagnosis of catamenial pneumothorax was made.

**Figure 1:**
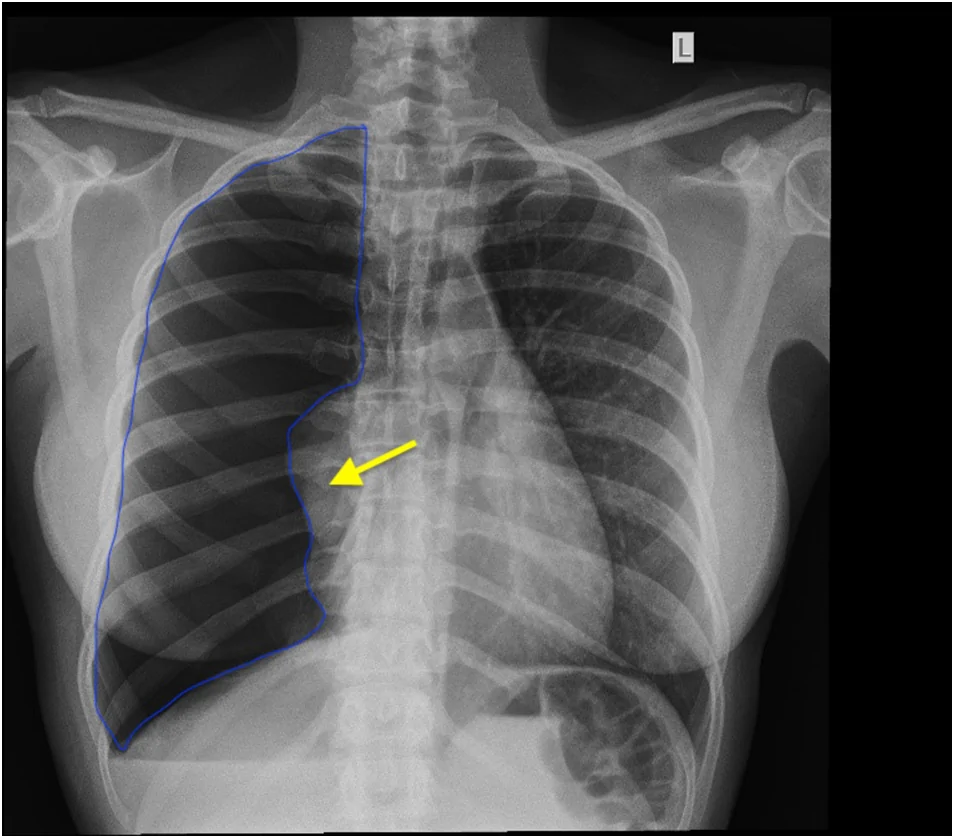
Chest radiograph (posteroanterior view) showing a massive right-sided pneumothorax (blue outline) with near-complete right lung collapse (yellow arrow).

### Therapeutic intervention (initial presentation)

The patient was admitted and managed by the cardiothoracic surgery team. A chest tube was inserted, and she commenced chest physiotherapy, including incentive spirometry. Serial chest radiographs showed progressive lung re-expansion and resolution of the pneumothorax, with corresponding clinical improvement. She was counselled regarding definitive surgical management but declined intervention. She was subsequently discharged with outpatient follow-up.

### Recurrence and definitive management

At the onset of her next menstrual period, she re-presented with similar symptoms. On examination, she was in respiratory distress, with a respiratory rate of 30 cycles per minute, pulse rate of 110 beats per min, blood pressure of 105/68 mmHg, and oxygen saturation of 93% in room air. Breath sounds were absent over the right hemithorax. Supplemental oxygen was commenced and an emergency needle decompression was performed till she was hemodynamically stable. A Chest x-ray and CT were subsequently obtained and revealed a right-sided tension pneumothorax (Figures 2, 4A).

**Figure 2:**
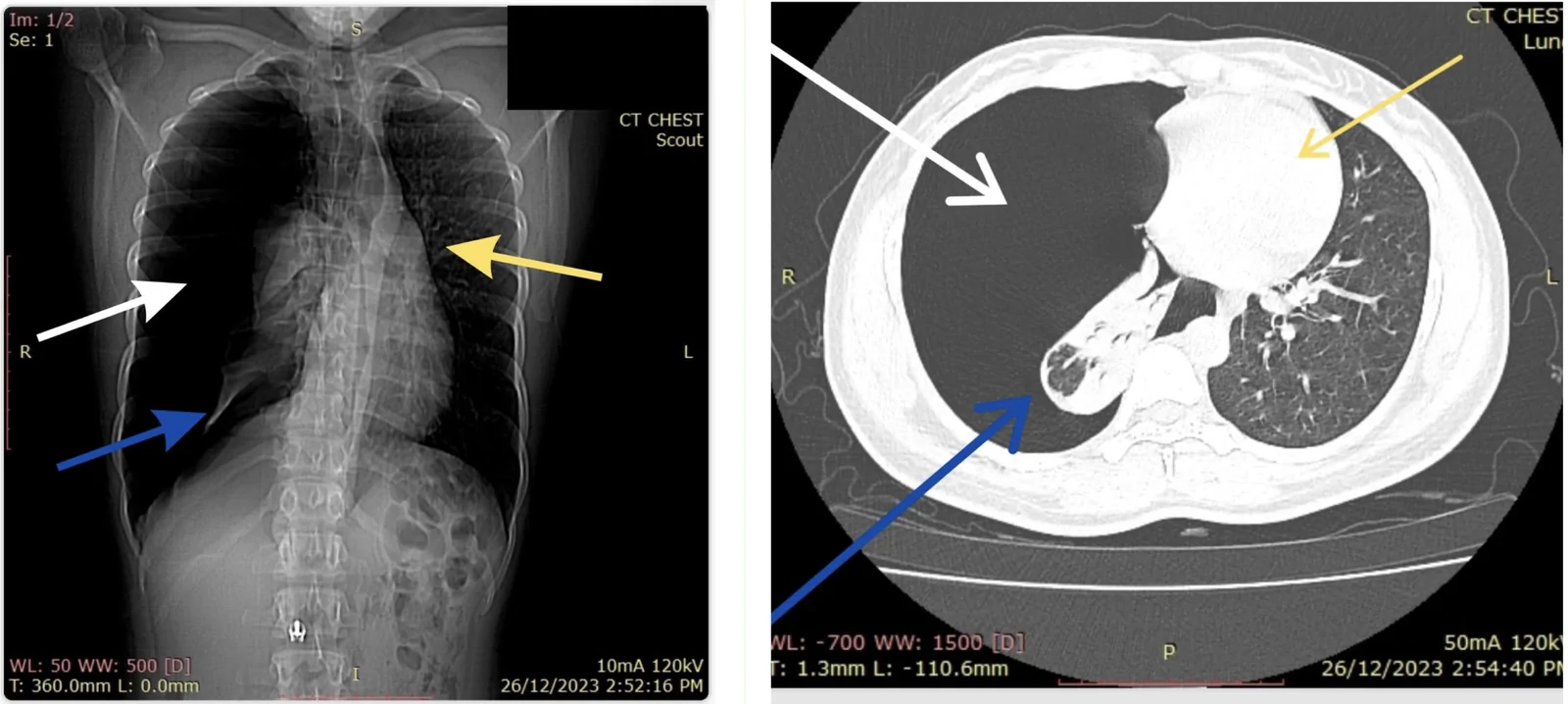
**(A)** Chest CT scout film and **(B)** Chest CT lung window (axial view) both showing right sided pneumothorax (white arrow), collapsed right lung (blue arrow) and mediastinal shift from pneumothorax (yellow arrow).

**Figure 3:**
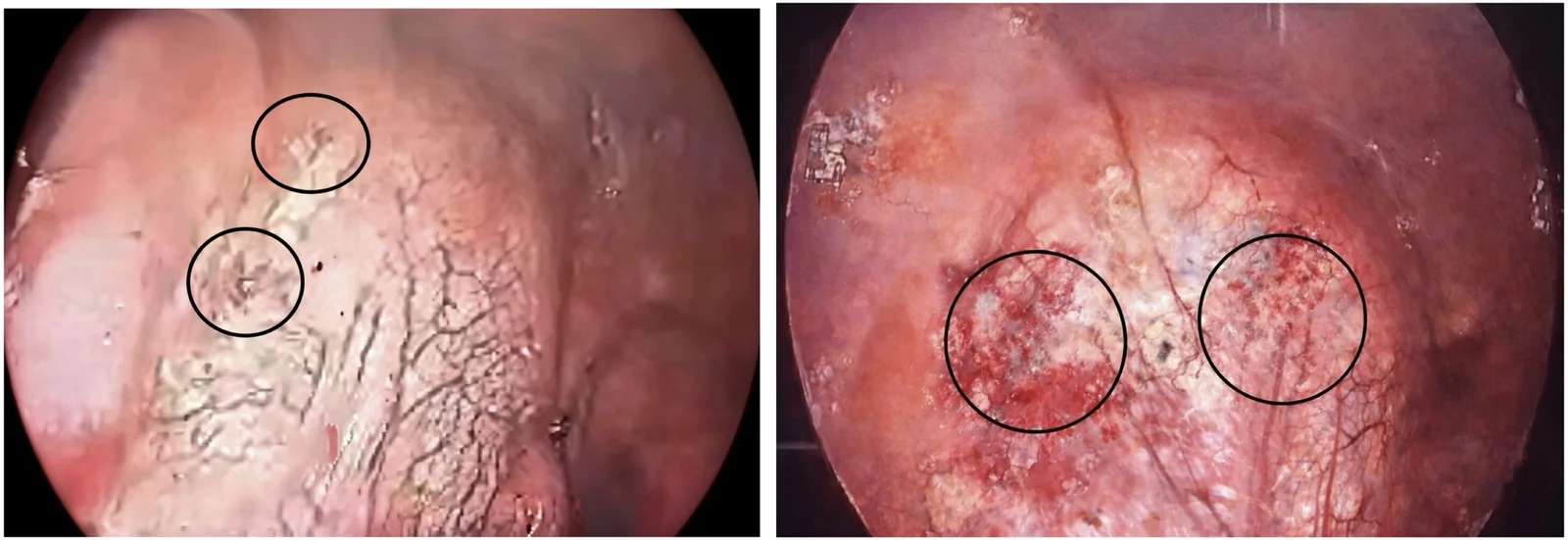
VATS images of the right hemidiaphragm demonstrating **(A)** multiple diaphragmatic perforations and **(B)** multiple endometriotic implants on the diaphragmatic pleural surface.

**Figure 4:**
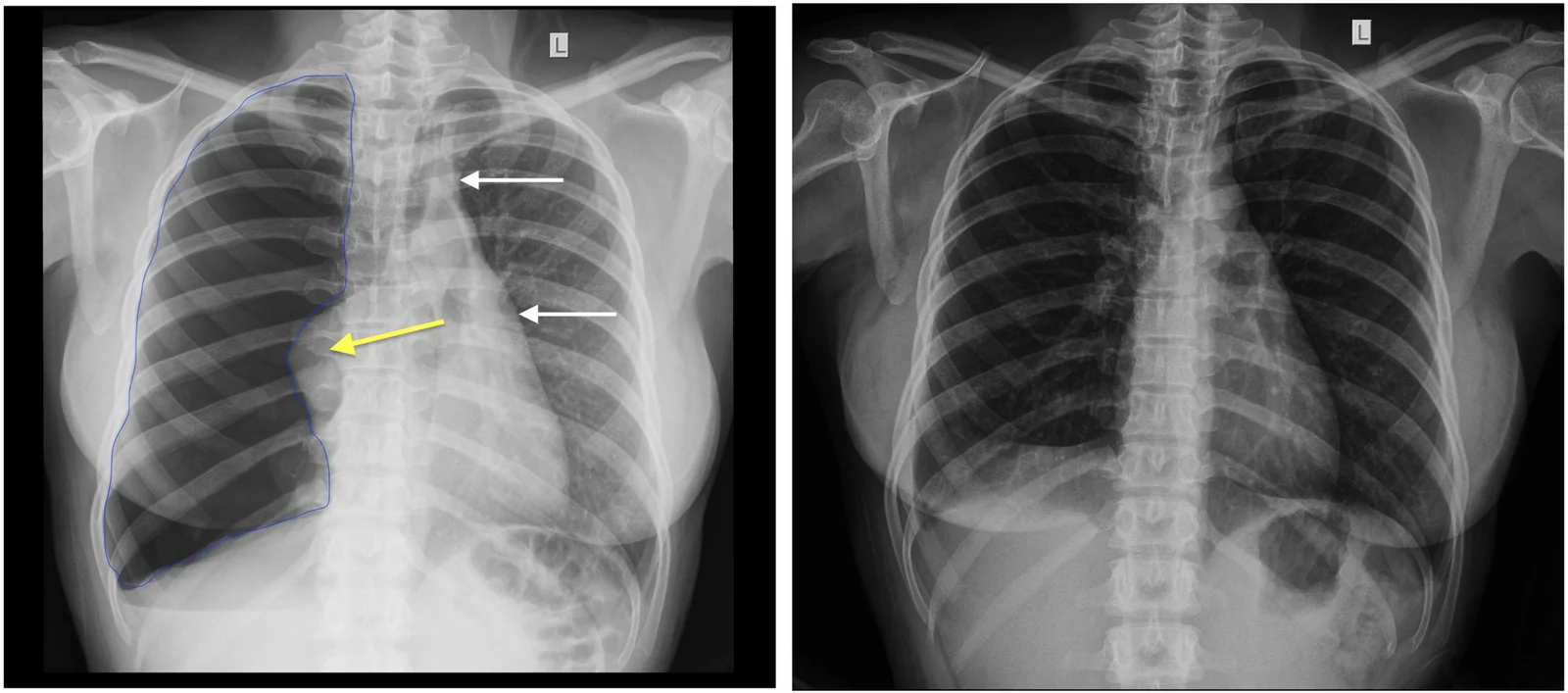
**(A)** Chest radiograph (posteroanterior view) at re-admission showing a massive right-sided pneumothorax (blue outline) with near-complete right lung collapse (yellow arrow) and mediastinal shift (white arrows) **(B)** chest radiograph showing resolution of the pneumothorax at discharge.

She thereafter underwent a chest tube re-insertion. She subsequently underwent video-assisted thoracoscopic surgery (VATS), a minimally invasive procedure allowing direct visualization and management of intrathoracic pathology. Intraoperatively, two diaphragmatic perforations measuring approximately 1 mm each, and multiple endometriotic implants were identified in the thoracic cavity, resected and sent for histopathological confirmation. Surgical repair of the diaphragmatic defects was performed by suturing, after which a mechanical pleurodesis was done.

Histopathology report confirmed fragments of fibrous tissue with congested vessels and areas of hemorrhage; fragments of endometrial stroma with a single gland seen and associated hemosiderin-laden macrophages. There were also foci of mesothelial hyperplasia with no atypia. These are features in keeping with endometriosis.

### Postoperative outcome and follow-up

Postoperative recovery was uneventful. Serial chest radiographs demonstrated sustained lung re-expansion (Figure 4B). Following several days of respiratory physiotherapy and clinical stabilization, she was discharged with follow-up in both cardiothoracic surgery and gynaecology clinics. Hormonal therapy was deferred after careful consideration of her reproductive plans and the potential temporary delay in conception associated with treatment. At the 2-month postoperative follow-up, she was evaluated by the gynecologist for new-onset dysmenorrhea. An abdominopelvic ultrasound was unremarkable; however, further investigations for pelvic endometriosis including an MRI and laparoscopy could not be performed because of financial constraints. She remained asymptomatic, with no recurrence of breathlessness or pneumothorax at 6 months after surgery. Thereafter, she was lost to follow-up. Timeline is shown in Table 1 above.

**Table 1:** Timeline.

Time point	Event
Day-3 (pre-menses)	Onset of chest tightness, dyspnoea, dizziness
Day 0	Presentation to hospital; diagnosed with right pneumothorax
Day 0–5	Chest tube insertion and conservative management; clinical improvement
Discharge	Patient declined definitive surgery
1 month later (next menses)	Recurrence with severe symptoms; tension pneumothorax diagnosed
Same day	Emergency chest tube insertion
Same admission	Underwent VATS with resection of endometriotic implants and diaphragmatic repair
Post-operative period	Uneventful recovery; lung re-expansion confirmed
2 months follow-up	New onset dysmenorrhea
6 months follow-up	No recurrence; patient asymptomatic

## Discussion

Endometriosis is a chronic oestrogen-dependent gynaecological condition defined by the presence of functional endometrial tissue and stroma outside the uterus. Although it predominantly affects pelvic structures, extra-pelvic involvement has been described in multiple sites, including the stomach, gallbladder, central nervous system, liver, kidneys, and lungs. Thoracic endometriosis represents one such extra-pelvic manifestation and may present clinically as catamenial pleuritic chest pain, haemoptysis, haemothorax, or pneumothorax. Catamenial pneumothorax (CP), defined as spontaneous pneumothorax occurring in temporal relation to menstruation, typically presents with chest pain and dyspnoea, as observed in our patient. A study conducted in France reported that women with endometriosis-associated pneumothorax most commonly presented with chest pain (90.5%), shortness of breath (72.9%), and cough (7.4%) (5). CP most commonly occurs in women aged 20–40 years and has a strong predilection for the right hemithorax. Similar cases reported in Ghana and Spain involved predominantly nulliparous women with right-sided pneumothorax who required surgical management (5, 6). Although CP is frequently associated with thoracic endometriosis (TE), its pathogenesis is not fully understood, and several alternative etiologic mechanisms have been proposed.

The physiologic hypothesis suggests that elevated prostaglandin F2 levels during menstruation cause vasoconstriction and bronchospasm, leading to alveolar rupture and pneumothorax, even in the absence of pelvic endometrial lesions. Increased prostaglandin levels may also render pre-existing pulmonary blebs more susceptible to rupture (3). The metastatic or lymphovascular microembolization theory proposes that endometrial tissue spreads via venous or lymphatic channels to the lungs, where necrosis of pleural-based implants can result in air leaks and pneumothorax, while centrally located lesions may cause haemoptysis (7).

Another explanation is the trans-genital trans-diaphragmatic passage of air theory, which suggests that air travels from the vagina through the uterus and fallopian tubes into the peritoneal cavity, and subsequently into the pleural space via diaphragmatic defects. These defects are typically right-sided, explaining the predominance of right-sided disease (3). Similarly, the migration theory proposes that retrograde menstruation leads to implantation of endometrial tissue in the pelvis, with subsequent migration to the sub-diaphragmatic region, particularly the right hemidiaphragm. This may result in diaphragmatic perforations and translocation of endometrial tissue into the thoracic cavity, ultimately triggering pneumothorax (7).

Although uterine leiomyomas have been reported to coexist with pelvic endometriosis (8), no direct causal relationship has been established between a history of myomectomy, as in our patient, and the development of either pelvic or thoracic endometriosis. The observed association between uterine leiomyomas and pelvic endometriosis has been attributed to factors such as uterine retroversion or distortion of the uterine cavity, which may facilitate retrograde menstruation and the implantation of ectopic endometrial tissue (8).

In contrast, abdominal wall endometriosis occurring after uterine surgery, particularly following cesarean section, is a well-recognized phenomenon (9, 10). It has been suggested that the endometrium during pregnancy possesses a microenvironment that favors ectopic implantation (9). Several mechanisms have been proposed to explain iatrogenic endometriosis, including retrograde menstruation, metaplasia, venous or lymphatic metastasis, and mechanical transplantation of endometrial tissue during surgery, with the latter being the most widely accepted mechanism (9).

Diagnosis of CP is often challenging and requires a high index of suspicion, particularly in women presenting with recurrent pneumothorax in relation to menstruation. Initial evaluation typically involves chest imaging, such as chest radiography, to confirm pneumothorax. However, definitive diagnosis often requires direct visualization. Video-assisted thoracoscopic surgery (VATS) plays a central role in the diagnosis and treatment of catamenial pneumothorax by enabling minimally invasive inspection of the thoracic cavity, identification of diaphragmatic defects and endometriotic lesions, and concurrent surgical management (11).

Management strategies for CP include both surgical and non-surgical approaches. Initial management of pneumothorax generally involves conservative measures such as needle decompression and chest tube drainage, as was performed in our patient. Surgical options include resection/cauterization of endometrial deposits, repair of diaphragmatic defects, and pleurodesis (mechanical or chemical) (3). Surgical repair may involve either a primary suture repair or mesh reinforcement. While several authors advocate diaphragmatic coverage with synthetic or biologic mesh to reinforce repaired defects and potentially reduce recurrence, particularly in patients with multiple or extensive diaphragmatic lesions, others have reported satisfactory outcomes with primary suture repair for small, localized fenestrations (12, 13). In our patient, the diaphragmatic fenestrations were small (approximately 1 mm each), and therefore primary suture repair was performed rather than diaphragmatic mesh reinforcement. Furthermore, although a recent practice, the use of biologic mesh has shown promising results in reducing recurrence, with one study recording a 0% recurrence during follow up (12).

Non-surgical management primarily involves hormonal suppression using gonadotropin-releasing hormone (GnRH) agonists such as leuprolide or continuous oral contraceptives to inhibit ectopic endometrial activity (3, 11). Continuous oral contraceptives may offer a long-term alternative with fewer hypoestrogenic side effects compared to GnRH agonists and may reduce the frequency of catamenial symptoms by preventing cyclic bleeding (3, 11).

Current treatment approaches increasingly advocate a multimodal approach which consists of thoracoscopic surgery combined with postoperative hormonal suppression, commonly using GnRH agonists or other ovulation suppressing therapies for approximately 6–12 months, while taking fertility goals and potential contraindications into account. This combined approach has been associated with improved outcomes and reduced recurrence rates (14).

A multidisciplinary approach should be adopted in the management of CP/TE, involving cardiothoracic surgeons, gynecologists, radiologists, pathologists, and, where appropriate, fertility specialists (15). Cardiothoracic surgeons play a pivotal role in the acute management of pneumothorax and definitive thoracic surgery, while gynecologists are essential for evaluating concomitant pelvic endometriosis which may contribute to recurrence if left untreated (4, 15). In women desiring future pregnancy, early involvement of fertility specialists enables individualized counselling regarding preservation of fertility and choice of treatment strategies that balance disease control with reproductive goals (16). A coordinated multidisciplinary assessment also facilitates combined thoracoscopic and laparoscopic management, enabling comprehensive treatment of thoracic and pelvic disease and potentially reducing recurrence (8, 15).

In the index patient, hormonal therapy was declined because she was nulliparous and wished to preserve her future fertility. Fertility preservation is an important consideration in the management of women of reproductive age with thoracic endometriosis. In many African settings, childbearing carries substantial social and cultural significance, which may further influence patients' acceptance of hormonal therapies. A single dose of GnRH agonist has been proposed as a potential option for women who wish to preserve their fertility. In one reported case, this approach delayed recurrence for 10 years (16).

Endometriosis is a recognized cause of subfertility, and concerns about the potential impact of hormonal therapy on future fertility may further increase the psychosocial burden experienced by affected women. Delayed diagnosis and recurrent episodes have also been shown to contribute to anxiety with each menstrual cycle, depression, emotional distress, and feelings of hopelessness, significantly reducing patients’ quality of life (16, 17).

Recurrence remains a major challenge in CP management. One study reported a median time to recurrence of 17.7 months following initial surgery, although this varies considerably according to the treatment modality employed (18). Postoperative recurrence rates range from 8% to 40%, while recurrence rates may exceed 50% in patients managed with hormonal therapy alone (3). Recent evidence from a systematic review and meta-analysis by Elsayed et al. demonstrated that adjunctive postoperative hormonal suppression was associated with substantially lower recurrence rates (approximately 17%) compared with surgery alone (>50%), although the available evidence still remains largely observational (14).

Several factors contribute to postoperative recurrence, including incomplete identification and resection of endometrial implants, failure to recognize sub-diaphragmatic lesions or fenestrations, the absence of adjuvant hormonal therapy, and untreated concomitant pelvic endometriosis (19). Diaphragmatic involvement is considered central to both the pathogenesis and recurrence of catamenial pneumothorax (20). Failure to identify and repair diaphragmatic fenestrations or resect diaphragmatic endometriotic implants during surgery has been associated with higher recurrence rates. Consequently, careful inspection of the diaphragm intraoperatively as well as repair of visible defects are regarded as crucial components of definitive surgical management (20).

Pelvic endometriosis coexists with thoracic endometriosis in up to 67.6% of affected women (4), highlighting the importance of multidisciplinary follow-up with a cardiothoracic surgeon, gynecologist, and other relevant specialists. Such collaboration facilitates early recognition of recurrent thoracic disease, assessment for concomitant pelvic involvement, and individualized long-term management (15, 16). Although the index patient had no pre-existing symptoms suggestive of pelvic endometriosis and transabdominal ultrasound was unremarkable, occult pelvic endometriosis could not be definitively excluded because of patient- and resource-related considerations. Consequently, it remains uncertain whether the patient's newly developed dysmenorrhea represented previously unrecognized pelvic endometriosis.

Symptom-based exclusion of pelvic endometriosis has its limitations. As high as 46% of women with CP/TE who were initially asymptomatic for pelvic disease were diagnosed after a pelvic MRI was done (4). Furthermore, transabdominal ultrasound has a relatively lower sensitivity of 81%, specificity of 38% and accuracy of 73% when compared to a pelvic MRI which has a sensitivity of 85%, specificity of 86% and accuracy of 85%, or even a diffusion weighted MRI with a sensitivity of 97%, specificity of 86% and accuracy of 95% in the diagnosis of pelvic endometriosis (21). Laparoscopic visualization and histopathological confirmation remain the gold standard for the diagnosis of pelvic endometriosis (22).

Although the clinical presentation in most cases of CP is often mild, it can occasionally present with severe complications such as tension pneumothorax, resulting in hemodynamic instability, as seen in this case. Such presentations are rarely reported. While a case of large right-sided tension pneumothorax associated with thoracic endometriosis was documented in the United States (23), to the best of our knowledge, this case represents the only report describing tension pneumothorax as a complication of CP in Africa, hence our decision to report it.

Our report, however, has some limitations. First, the relatively short duration of follow-up may not adequately reflect the long-term risk of recurrence, particularly because the patient declined postoperative hormonal therapy, which has been shown to reduce recurrence rates (14). In addition, financial constraints prevented further evaluation for pelvic endometriosis with diagnostic laparoscopy.

While VATS remains the cornerstone for the diagnosis and surgical management of thoracic endometriosis through resection of visible lesions and pleurodesis, its visualization is limited to the pleural surface of the diaphragm and may fail to detect lesions located on the peritoneal (subdiaphragmatic) surface. D’Ancona et al. reported the coexistence of subdiaphragmatic and thoracic endometriosis in 2.2% of patients, showing the possibility of occult subdiaphragmatic disease (24). Consequently, combined VATS and diagnostic laparoscopy is recommended when feasible to enable comprehensive evaluation of both the thoracic and abdominopelvic cavities (25). Liver mobilization during laparoscopy has also proved important in excluding involvement of the posterior diaphragm (26).

Taken together, the absence of postoperative hormonal therapy, the inability to definitively exclude occult pelvic and subdiaphragmatic endometriosis, and the patient's subsequent loss to follow-up all increase the uncertainty regarding her long-term prognosis. These factors may place her at an elevated risk of recurrence while also limiting long-term surveillance and timely detection of recurrent disease.

## Conclusion

This case highlights catamenial pneumothorax presenting as tension pneumothorax, a rare but potentially life-threatening manifestation that requires a high index of suspicion for timely diagnosis and management. It explains the importance of a multidisciplinary approach to management. It also illustrates the challenges encountered in low-resource settings, where financial constraints may limit comprehensive evaluation, and sociocultural concerns regarding fertility may limit acceptance of postoperative hormonal therapy. Given the significant risk of recurrence, long-term follow-up and surveillance are essential to detect recurrent disease.

## Data Availability

The original contributions presented in the study are included in the article/Supplementary Material, further inquiries can be directed to the corresponding author.
